# Effects of MicroRNA-23a on Differentiation and Gene Expression Profiles in 3T3-L1 Adipocytes

**DOI:** 10.3390/genes7100092

**Published:** 2016-10-24

**Authors:** Yong Huang, Jinxiu Huang, Renli Qi, Qi Wang, Yongjiang Wu, Jing Wang

**Affiliations:** 1 Chongqing Academy of Animal Science, Rongchang, Chongqing 402460, China; xuehai12@tom.com (Y.H.); wangq0418@126.com (Q.W.); wj57482199@163.com (J.W.); 2 Key Laboratory of Pig Industry Sciences, Ministry of Agriculture, Rongchang, Chongqing 402460, China; short00@163.com; 3 College of Animal Science and Technology, Southwest University Rongchang Campus, Rongchang, Chongqing 402460, China; Wyongjang@163.com

**Keywords:** microRNA-23a, adipocytes, differentiation, DGE-Seq, differentially expressed genes

## Abstract

MicroRNAs (miRNAs) are small non-coding RNA molecules that regulate growth, development, and programmed death of cells. A newly-published study has shown that miRNA-23a could regulate 3T3-L1 adipocyte differentiation. Here, we identified miRNA-23a as a negative regulator of 3T3-L1 adipocyte differentiation again. Over-expression of miRNA-23a inhibited differentiation and decreased lipogenesis as well as down-regulated mRNA and protein expression of both peroxisome proliferator-activated receptor (PPAR) γ and fatty acid binding protein (FABP) 4, whereas knock down of miRNA-23a showed the opposite effects on differentiation as well as increasing the number of apoptotic cells. Additionally, digital gene expression profiling sequencing (DGE-Seq) was used to assay changes in gene expression profiles following alterations in the level of miR-23a. In total, over-expression or knock down of miRNA-23a significantly changed the expression of 313 and 425 genes, respectively. Gene Ontology (GO) and Kyoto Encyclopedia of Genes and Genomes (KEGG) analyses indicated that these genes were mainly involved in the stress response, immune system, metabolism, cell cycle, among other pathways. Additionally, the signal transducer and activator of transcription 1 (Stat1) was shown to be a target of miRNA-23a by computational and dual-luciferase reporter assays that indicated Janus Kinase (Jak)-Stat signal pathway was implicated in regulating adipogenesis mediated by miRNA-23a in adipocytes.

## 1. Introduction

MicroRNAs (miRNAs) are small non-coding RNAs between 18 and 25 nucleotides in length that are present in all eukaryotes [1]. MicroRNAs have complex and diverse regulatory functions in cells through fine control of a large number of targeted genes. They bind to the 3′ untranslated region of targeted genes via base pairing with complementary sequences and then recruit the RNA-induced Silencing Complex (RISC) that either degrade the target messenger RNA (mRNA) or inhibit its translation [2,3]. Usually, a given miRNA has up to thousands of targets implicated in the regulation of cellular growth, development, and death.

Recently, miRNA-23a (miR-23a) has received increased attention because of the diverse group of cell types in which it has important regulatory functions. The microRNAs miR-23a, miR-27a, and miR-24-2 constitute a co-expressed miRNA cluster located in an intergenic region in vertebrates [4,5]. Because miR-23a is highly expressed in many tumor cells, it is regarded as a molecular marker for cancer progression. In previous studies, miR-23a exhibited different effects on the proliferation, differentiation, and apoptosis of many different tumor cells [6,7,8,9,10]. Moreover, additional studies indicate that miR-23a is involved in the control of cardiac function [11,12], osteal formation [5,13], and immunoreactions [14,15] in both humans and animals. In addition, miR-23a is also expressed in adipocytes. Recently, Shen and colleagues found miR-23a regulated 3T3-L1 cell differentiation via decreasing mRNA levels of adipocyte specific genes involved in lipogenic transcription, fatty acid synthesis and fatty acid transport [16]. However, details about the regulatory function of miR-23a and targets are not entirely known.

In this study, synthetic miRNA mimics and inhibitors were used in gain- and loss-of-function experiments to investigate the roles of miR-23a in 3T3-L1 cells which are a widely-used fundamental model in the study of adipocytes. Then, digital gene expression profiling sequencing (DGE-Seq), a powerful tool [17,18] for analyzing high-throughput gene expression datasets, was used to identify changes in gene expression as a result of altered miR-23a. We found that over-expressed miR-23a significantly inhibits adipogenic differentiation and decreases lipid deposition in 3T3-L1 cells, whereas knockdown of miR-23a promotes differentiation and apoptosis of cells. Hundreds of genes showed differential expression owing to altered miR-23a levels. Gene Ontology (GO) and Kyoto Encyclopedia of Genes and Genomes (KEGG) analysis indicated that most of the changed genes were related to the immune system and stress response. Moreover, altered miR-23a levels interfere with the expression of signal transducer and activator of transcription 1 (Stat1) and then it was predicated and confirmed for a target gene for miR-23a. Therefore, miR-23a may control adipogenesis in adipocytes through the Janus Kinase (Jak)-Stat signaling pathway. These findings will help us to have a better understanding about the regulatory function of miR-23a and the peculiar metabolism of adipocytes.

## 2. Materials and Methods

### 2.1. Cell Culture

The 3T3-L1 preadipocytes were seeded in Dulbecco’s modified Eagle’s medium/F12 (Gibco, Grand Island, NY, USA) supplemented with 10% fetal bovine serum and cultured in 6-well plates at 37 °C under a humidified 5% CO_2_ atmosphere. To induce adipogenic differentiation, 0.5 mM 3-isobutyl-1 methylxanthine, 1 µM dexamethasone, and 5 µg/mL insulin (Sigma-Aldrich, St. Louis, MO, USA) was added to the culture medium after two days of fusion of the cells followed by 5 µg/mL insulin-only treatment for another two days. After treatment, the inducers were removed from the culture medium.

### 2.2. RNA Oligonucleotides and Transfection

The miR-23a mimics, inhibitors of miR-23a, and native control oligos were custom synthesized by the RIBOBIO Company (Guangzhou, China). During the second day of differentiation, the mimics or inhibitors (final concentration 100 nM) were transfected into cultured cells for over-expression or knockdown of miR-23a, respectively. Total RNA was extracted 48 h post-transfection for quantitative PCR (qPCR) and total protein was extracted 72 h post-transfection for a caspase 3/7 activity assay and western blotting.

### 2.3. Oil Red O Staining

Progression of adipogenesis in 3T3-L1 cells was determined by Oil Red O staining. Briefly, cells were collected at the 10th day of differentiation, washed three times with phosphate-buffered saline (PBS) (pH 7.2), and then fixed in 4% paraformaldehyde for 30 min. After washes, the fixed cells were stained with a working solution of Oil Red O (60% Oil Red O stock solution consisting of 0.5% Oil Red O in isopropanol and 40% H_2_O) for 30 min at room temperature. Lastly, the cells were washed with deionized water, and the stain was extracted by using 100% avantin for colorimetric analysis at 510 nm (quantitative analysis of triglyceride).

### 2.4. Caspase3/7 Activity Analysis

A Caspase-Glo^®^ 3/7 Assay Kit (Promega, Los Angeles, CA, USA) was used to quantitate caspase 3/7 activity according to the manufacturer’s instructions. Briefly, 100 µL of Caspase-Glo^®^ 3/7 reagent was added to each well of a 96-well plate containing 100 µL of blank, negative control cells, or treated cells and then incubated at room temperature for 1 h. Fluorescence intensity was detected by using a fluorescence microplate reader (Synergy™ 4; Biotek, Winooski, VT, USA) and caspase 3/7 activity was determined using the fluorescence intensity.

### 2.5. Terminal-Deoxynucleotidyl Transferase-Mediated Nick End Labeling (TUNEL) Assay

Apoptosis in adipocytes was measured using a DeadEnd™ Fluorometric TUNEL System (Promega). Briefly, cultured adipocytes or frozen sections of mouse fat tissue were fixed with 4% neutral formalin for 15 min and then the samples were washed with PBS and permeabilized with Proteinase K. After washing, the samples were immersed in incubation buffer containing equilibration buffer, nucleotide mix, and rTdT Enzyme at 37 °C for 1 h. The slides were dipped in 2× Saline Sodium Citrate (SSC) to stop the reaction. Cells were stained for 5 min with an aqueous solution of 4',6-Diamidino-2-phenylindole (DAPI) (10 µg/mL) to visualize nuclei. Finally, green fluorescence of apoptotic cells (fluorescein-12-dUTP) in a blue background (DAPI) was observed using fluorescence microscopy.

### 2.6. Digital Gene Expression Profiling Sequencing (DGE-Seq)

Total RNA was extracted from the cells in different groups using Trizol reagent (Invitrogen, San Diego, CA, USA) and RNA quantity and purity were determined using a Bioanalyzer 2100 with an RNA 6000 Nano LabChip Kit (Agilent, Santa Clara, CA, USA). All samples had an RNA integrity number greater than 7.0. For each sample, poly (A) plus mRNA was isolated from approximately 10 µg of total RNA using poly T oligos attached to magnetic beads (Thermo-Fisher, Waltham, MA, USA). Following purification, the purified mRNA was fragmented into small pieces using divalent cations under elevated temperature. Then, the cleaved RNA fragments were used to generate a cDNA library for sequencing following the protocol for the Illumina RNA ligation based method (Illumina, San Diego, CA, USA).

The raw data containing adaptor sequences, tags with low-quality sequences, and unknown nucleotides was filtered to obtain clean reads 36 nt in length. Clean reads were then subjected to quality assessment. All clean tags were aligned to annotated transcripts using Bowtie, with only one mismatch allowed per aligned read. To identify mapping events in both the sense and anti-sense orientations, both the sense and the complementary antisense transcript sequences were included in the reference data collection. The number of perfect clean reads corresponding to each gene was calculated and normalized to the reads per kilobase of exon model per million mapped reads. Based on the expression levels, the significant differentially expressed genes (DEGs) among different samples were identified with *p* value < 0.05 and log2 fold-change (log2 FC) ≥1. Clustering of DEGs was performed using common Perl and R scripts. GO analysis was conducted for functional classification of DEGs and the pathway analysis was carried out using KEGG.

### 2.7. RNA Extraction and qRT-PCR

Mature miR-23a in the cells were detected by poly (A)-tailed qRT-PCR. Briefly, small RNA was extracted from cultured cells with Small RNA Reagent (TaKaRa, Dalian, China) and initially reverse transcribed and amplified with gene-specific primers using a SYBR^®^ PrimeScript™ miRNA RT-PCR kit (Clontech, Mountain View, CA, USA). 5S rRNA served as an endogenous reference RNA or normalizing miR-23a expression. Sequences of qRT-PCR primers are showed in the Table S1.

For detection of mRNA expression levels, total RNA was extracted from cultured cells using RNAiso Reagent (TaKaRa, Japan), and 1.0 μg samples were reverse transcribed to complementary DNA (cDNA) using a PrimeScript™ RT reagent Kit (TaKaRa). PCR was performed using a Step One system (ABI, NY, USA) with SYBR Premix Ex Taq™ II (TaKaRa). The 18S rRNA and glyceraldehyde-3-phosphate dehydrogenase (GAPDH) were used as reference genes with similar results.

### 2.8. Protein Isolation and Western Blotting

Cells were collected at the specified times and lysed in radio immunoprecipitation assay (RIPA) buffer (Beyotime, Beijing, China). Lysates were analyzed using a standard western blot procedure. The β-tubulin and GAPDH antibodies were used as loading control. Peroxisome proliferator-activated receptor (PPAR) γ and fatty acid binding protein (FABP) 4, and Stat1 antibodies and horseradish peroxidase conjugated secondary antibodies were obtained from CST (Danvers, MA, USA).

### 2.9. Target Prediction and Dual-Luciferase Activity Assay

The Targetscan target prediction online tool showed there was a binding site in the 3’-UTR of *Stat1* gene. The 3’- untranslated regions (UTR) of *Stat1* containing an intact miR-23a recognition sequence (280 bp) was cloned into the pGL3-control-mcs2 reporter vector (constructed by our laboratory). Oligonucleotides (300 bp) harboring miR-23a binding sites from the mouse *Stat1* 3’-UTR were annealed and ligated into the EcoR I and Pst I sites of the pGL3-control mcs2 reporter vector. For the luciferase assays, the 293T cells were transfected with the appropriate plasmids in 24-well plates and harvested for luciferase activity assays using the dual-luciferase reporter assay system (Promega) at 48 h after transfection. The relative luciferase activity was normalized to that of firefly luciferase.

### 2.10. Statistical Analyses

All statistical analyses for data were performed using SPSS 19.0 software (IBM, New York city, NY, USA). The effects of the treatments were determined using the one-way analysis of variance. Comparisons among the means of individual treatments were made by the Fisher’s least significant difference. Data are reported as the mean ± the standard error of the mean and *p* < 0.05 was considered significant.

## 3. Results

### 3.1. Effects of Over-Expression and Knockdown of miR-23a on 3T3-L1 Adipocytes

**Figure 1:**
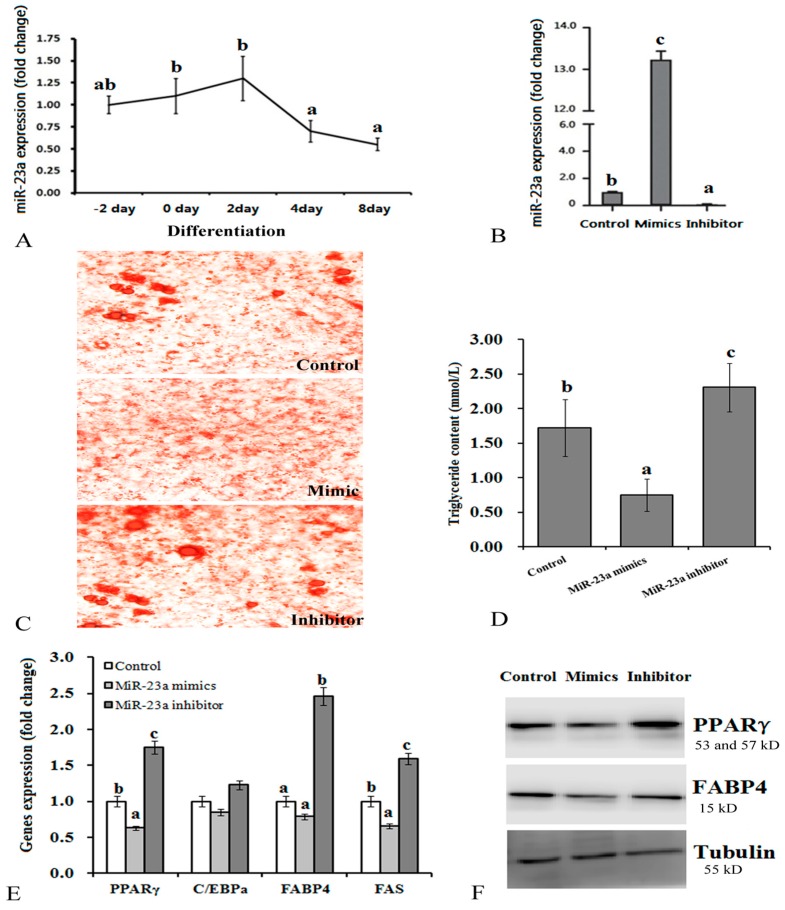
Effects of over-expression or knock down of miR-23a on differentiation of adipocytes. (**A**) Expression profiles of miR-23a during differentiation of 3T3-L1 adipocytes; (**B**) Over-expression and knock down of miR-23a by transfection of specific mimics and inhibitors in adipocytes; (**C**) Observation of Oil Red O-stained adipocytes (200× magnification); (**D**) Triglyceride content in adipocytes; (**E**) mRNA expression levels of genes related in fat synthesis. Levels of mRNA were determined by qRT-PCR; (**F**) Changes of peroxisome proliferator-activated receptor (PPAR) γ and fatty acid binding protein (FABP) 4 protein levels. *n* = 6, lowercase letters indicate significant differences between groups (*p* < 0.05).

Firstly, we determined the expression profile of miR-23a during the differentiation process of 3T3-L1 adipocytes by qRT-PCR. The data indicated that miR-23a was highly expressed at the early stage of differentiation (the 2nd day) and then gradually decreased throughout differentiation (Figure 1A). Next, we explored the effects of miR-23a expression levels on adipogenic differentiation of 3T3-L1 adipocytes by transfection of either miR-23a mimic or inhibitor. Compared with the control, mimicked transfection increased the expression of miR-23a by 12.5-fold (Figure 1B), significantly inhibited differentiation, and decreased production and accumulation of cellular lipids (Figure 1C,D). In addition, transfection of miR-23a mimic down-regulated expression of several adipogenesis factors (PPARγ, CCAAT-enhancer-binding protein (C/EBP)α, FABP4, and fatty acid synthase (FAS)) to different extents (Figure 1E,F). Conversely, transfection of miR-23a inhibitor decreased expression of miR-23a by 94% (Figure 1B) compared to the control. This accelerated differentiation and increased cellular lipid accumulation (Figure 1C–F).

**Figure 2:**
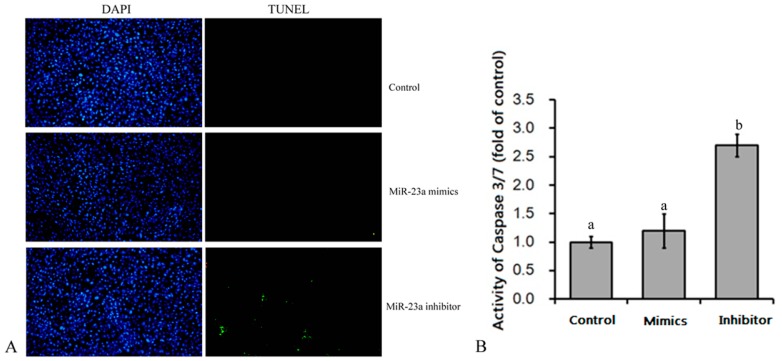
Effects of over-expression or knock down of miR-23a on apoptosis of adipocytes. (**A**) Terminal-deoxynucleotidyl transferase-mediated nick end labeling (TUNEL) analysis showed that knockdown of miR-23a increased the number of apoptotic cells after inhibitor transfection. The spots with green fluorescence in the figure indicate apoptotic cells; (**B**) Caspase 3/7 activity was higher in miR-23a knockdown cells than in control and miR-23a over-expressing cells. *n* = 6, lowercase letters indicate significant differences (*p* < 0.05).

We found that the number of apoptotic cells increased after inhibitor transfection (Figure 2A). Additionally, the activity of caspase 3/7 (Figure 2B), a key apoptotic factor, was increased in cells treated with the inhibitor relative to mimic-treated and control cells. This suggests that miR-23a deficiency increases apoptosis of 3T3-L1 adipocytes.

### 3.2. DGE-Seq Analysis of Differently Expressed Genes Caused by miR-23a

To identify genes whose expression changes in response to miR-23a, RNA libraries were generated from cells over-expressing miR-23a, cells knocked down for miR-23a, and control cells. Then, 9.01, 10.56, and 9.73 million raw reads from the libraries, respectively were produced by sequencing (Table 1). The tags were filtered for low-quality tags, tags with only one copy, and tags containing ambiguous nucleotides, which resulted in approximately 8.99, 10.54, and 9.71 million clean sequence tags, respectively. For the three libraries, 74.64%, 76.31%, and 80.04% of the clean tags were mapped unambiguously, with 2,449,235 (27.23% of the clean tags), 2,914,600 (27.66% of the clean tags), and 2,794,648 (28.81% of the clean tags) clean reads being perfectly mapped within the 16-nucleotide tag alignments, respectively (≤1 mismatch). Lastly, 19,828; 20,267, and 20,104 unique genes representing the DGE tags from the three libraries, respectively, were obtained, and the counts for each unique gene were normalized to the reads per kilobase of million reads (RPKM).

**Figure 3:**
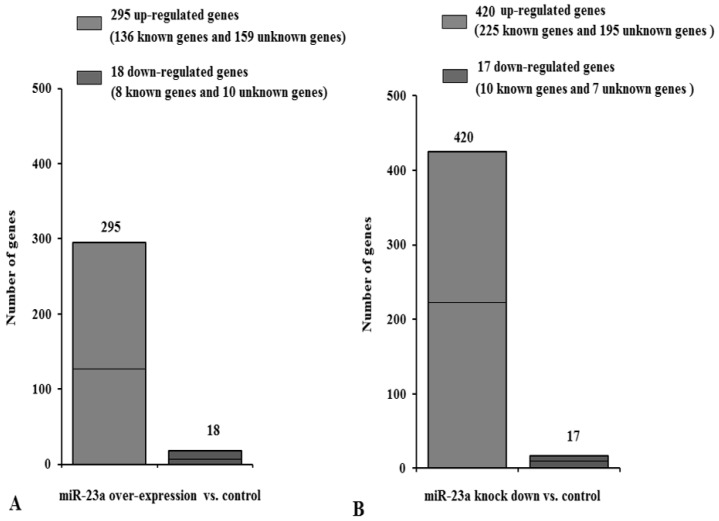
Number of differentially expressed genes following the altered miR-23a. (**A**) The number of differentially expressed genes identified in miR-23a over-expressing cells versus control cells. (**B**) The number of differentially expressed genes in miR-23a knockdown cells versus control cells.

A statistical analysis of the genes with observed fold changes determined by DGE analysis identified 313 genes (change fold > 2 fold, *p* < 0.05) that showed changed expression following over-expression of miR-23a (Figure 3A). Of these, 295 genes (including 136 genes of known function and 159 genes of unknown function) exhibited increased expression and 18 genes (including eight function known genes and ten function unknown genes) exhibited decreased expression. In contrast, miR-23a knock down (Figure 3B) resulted in 420 genes with increased expression and 17 genes with decreased expression (fold change > 2-fold, *p* < 0.05). Supplemental Table S1 indicates the genes with the biggest fold change.

### 3.3. GO and KEGG Analysis of Differently Expressed Genes

Prediction of the functions of DEGs (excluding genes with “unknown function”) was performed by using GO categories and KEGG pathway analysis.

**Figure 4:**
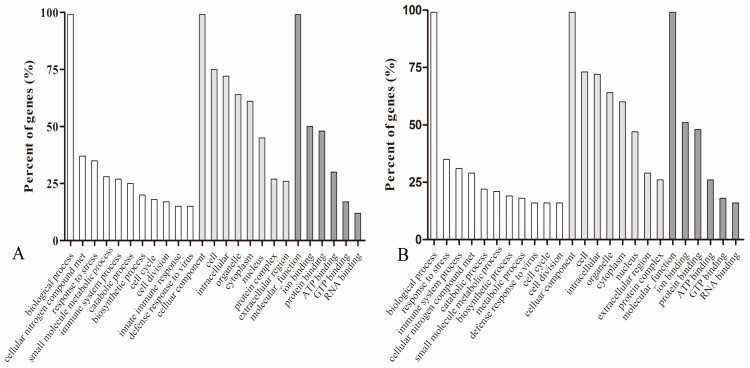
Gene Ontology (GO) analysis of genes differentially expressed as a result of altering miR-23a levels. (**A**) GO analysis of the differentially expressed genes as a result of miR-23a over-expression; (**B**) GO analysis of the differentially expressed genes as a result of miR-23a knock down.

Based on GO analysis, the DEGs caused by altered miR-23a expression levels were enriched for ten biological processes. It was noteworthy that four of the identified processes were involved in immunoreactions and stress response, including immune system process, response to stress, innate immune response, and defense to virus (Figure 4). This suggested miR-23a had an important regulatory function in cellular immunoreactions that is in agreement with several previous studies. In addition, the predicted major functional categories of DEGs were also implicated in cellular nitrogen metabolic processes, catabolic processes, small molecule metabolic processes, the cell cycle, cell division, and biosynthetic processes.

**Figure 5:**
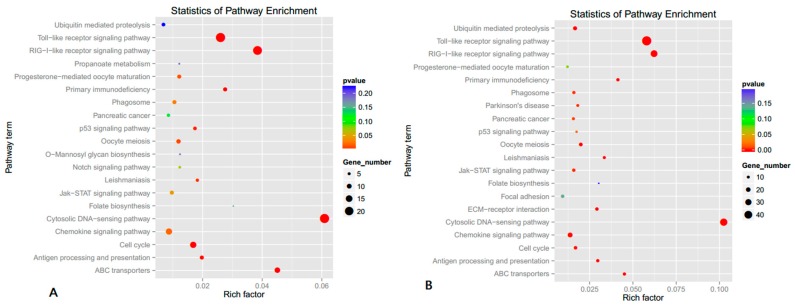
Kyoto Encyclopedia of Genes and Genomes (KEGG) assay for differentially expressed genes as a result of altered miR-23a expression. (**A**) KEGG assay for differentially expressed genes in miR-23a over-expressing cells versus control cells; (**B**) KEGG assay for differentially expressed genes in miR-23a knockdown cells versus control cells.

Next, we compared the DEGs with the KEGG pathway database to predict which biological pathways our identified genes participate in. Genes with differential expression in the presence of over-expressed miR-23a were enriched in 14 pathways (Figure 5A, *p* < 0.05) while genes with differential expression when miR-23a is reduced were enriched in 17 pathways (Figure 5B, *p* < 0.05). It was worth noting that, similar to the results of GO term analysis, KEGG analysis also suggested that miR-23a influenced the cellular immunoreaction and defense responses. In support of this, two of the biggest groups of DEGs as a result of altered miR-23a expression were associated with the RIG-I-like and Toll-like receptor pathways, which were both key modulators of the immune system and stress response.

### 3.4. *Stat1* Is a Target Gene of miR-23a

**Figure 6:**
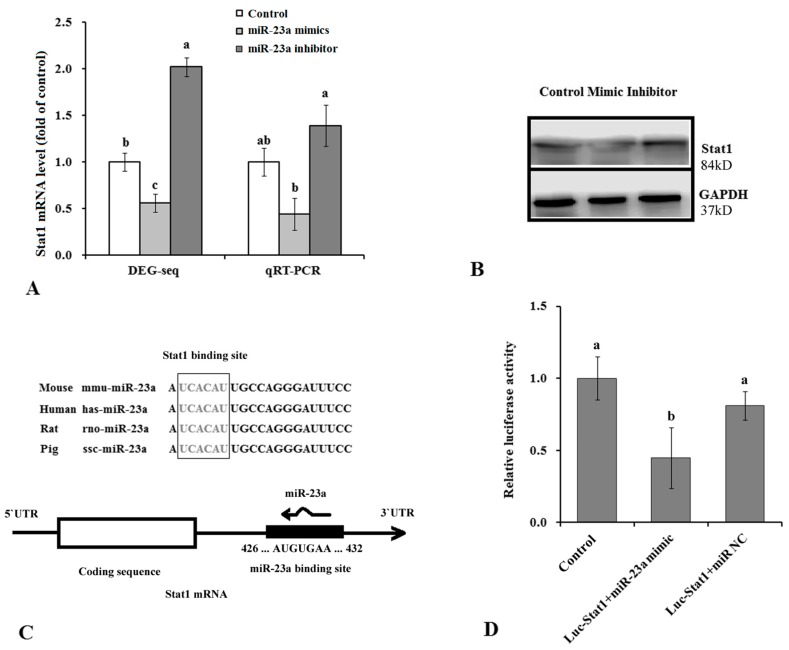
*Stat1* is a target gene of miR-23. (**A**) mRNA level of Stat1 was determined by the digital gene expression (DGE) sequencing and qRT-PCR. (**B**) Protein level of Stat1 was detected by western blotting assays. (**C**) The location of Stat1 that miR-23a binds to, and a magnified image. (**D**) Dual luciferase assay for miR-23a and target gene *Stat1*. Firefly luciferase activity in different treatments was tested following standard instruction, and corrected by Renilla luciferase. *n* = 6, lowercase letters indicate significant differences (*p* < 0.05). Luc: luciferase; NC: negative control.

Our KEGG analysis indicated that altered miR-23a expression also significantly affected the Jak-Stat signaling pathway (Figure 5, *p* < 0.01). It was noteworthy that the DGE sequencing data demonstrated that Stat1 was down-regulated by over-expressed miR-23a and up-regulated by knock down of miR-23a (Figure 6A). This result was further verified by qRT-PCR and western blotting assays. We found that mRNA and protein levels of Stat1 in the adipocytes were decreased by the miR-23a mimic and increased by the by the miR-23a inhibitor (Figure 6A,B). Moreover, taking advantage of the target gene predication tool, we found there was a conservative miR-23a binding site in the 3’UTR of Stat1 mRNA (Figure 6C) that suggested Stat1 was a direct target for miR-23a. A dual-luciferase reporter assay was carried out to verify the prediction and then the data showed that over-expression of miR-23a impaired the Firefly luciferase expression significantly when compared with control, which meant miR-23a could complimentarily pair that piece of 3’UTR sequence from Stat1 mRNA (Figure 6D). This confirmed *Stat1* gene was a real target for miR-23a. Since Stat1 was an important constituent element for the Jak-Stat pathway which was directly involved in the differentiation and adipogenesis of adipocytes, we believe that miR-23a regulated differentiation and adipogenesis in adipocytes partly by targeting Stat1.

## 4. Discussion

As a constituent element of adipose tissue, adipocytes are the major site of storage of triglycerides and an important fuel source [19]. As the incidence of human obesity, diabetes mellitus, and cardiovascular diseases increases, understanding the regulatory mechanisms underlying adipose tissue becomes more critical. Given the important regulatory functions of miRNAs in gene expression, exploring the role of miRNAs in the regulation of lipid metabolism may contribute to our understanding of these diseases. To date, dozens of miRNAs have been shown to be expressed in adipocytes [20,21]. Let-7 [22], miR-143 [23], miR-103 [24], miR-199a [25], and additional miRNAs exerted fine control over proliferation, differentiation, and lipid metabolism in adipocytes with the number of miRNAs implicated in the control in adipocytes still increasing.

Most previous studies of miR-23a focused on its functions in tumor cells because miR-23a expression was strongly correlated with growth and transformation of many types of tumor cells, suggesting that it was a biomarker for cancer. Additionally, it was believed that miR-23a also participated in the regulation of cardiomyogenesis, osteal development, and muscle growth [26]. However, thus far, we had only limited knowledge about its expression and function in adipocytes.

In a similar manner to the previous study [16], in our study it was observed that miR-23a was a negative regulator in the differentiation of 3T3-L1 adipocytes. Over-expressed miR-23a significantly decreased adipogenesis, which was likely owing to down-regulation of PPARγ and FAS. Conversely, knock down of miR-23a accelerated differentiation through up-regulation of PPARγ and FAS. In addition, one member of the miR-23a-miR-27-miR-24 miRNA cluster, miR-27a, was required for the differentiation of adipocytes [27,28]. It suppressed PPARγ expression, which results in inhibition of adipocyte differentiation. These findings suggested that the three miRNAs in the miRNA cluster may have similar expression patterns and similar regulatory functions in adipocytes. We additionally found that miR-23a deficiency increased the number of apoptotic cells compared with control. This increase correlated with an increase in caspase 3/7 activity. This indicated that normally expressed miR-23a is necessary for adipocytes and cells lacking it are more likely to undergo apoptosis. Additional studies of tumor cells also reported that abnormal expression of miR-23a affects cellular apoptosis [29,30]. There was a small difference between our present study and the previous study. Our result indicated that miR-23a is highly expressed at the initial stage of differentiation (0–2 days) and then follows a rapid down-regulation of expression. However, Shen [16] reported that the expression level of miR-23a was significantly down-regulated after the differentiation induction of 3T3-L1 cells. This difference may be caused by different detection or data statistical analysis method.

Typically, a single miRNA directly influences hundreds of targeted genes, which makes it challenging to fully explain the role of any individual miRNA. In the present study, we comprehensively profiled gene expression in response to altered miR-23a levels in adipocytes using the DGE method, a powerful tool for studying high-throughput gene expression profiles. Not surprisingly, hundreds of genes showed significant changes following over-expression or knockdown of miR-23a. Perhaps some were direct targets of miR-23a, while others were indirectly affected. GO and KEGG analyses suggested that most of the DEGs are related to immunoreactions, stress response, metabolism, and regulation of the cell cycle. This further demonstrates that miR-23a has intricate effects on the normal growth and development of cells.

Our DGE analysis and KEGG analysis revealed that the expression of Jak-Stat pathway components was changed following changes in the expression level of miR-23a. The Jak-Stat signaling pathway was a complex cascade that transduces many signals for development and homeostasis in animals [31]. In recent years, the Jak-Stat pathway has been determined to play a role in lipid metabolism in fat cells [32,33]. As an important member of the Stat protein family, Stat1 promotes adipogenesis and inhibited lipolysis in adipocytes by affecting expression of PPARγ and lipoprotein lipase [34,35]. Results of our study indicated that *Stat1* was a direct target gene for miR-23a and it was reduced by over-expressed miR-23a when knockout miR-23a exported an opposite effect on the gene. Additionally, Stat1 expression was increased while miR-23a expression was decreased through 3T3-L1 differentiation [34]. This implies that the Jak-Stat signaling pathway transduces the effects of miR-23a in adipocytes. Similarly, a recent paper also reported miR-23 targets Jak-Stat pathways in prostate cancer through computational and in vitro studies [36].

## 5. Conclusions

This study reveals that miR-23a is a negative regulator of differentiation and adipogenesis of adipocytes. However, cells with reduced levels of miR-23a are more likely to undergo apoptosis. Furthermore, DGE-Seq and bioinformatics analysis indicates that miR-23a influences immunoreactions and stress response in cells. More altered genes are enriched in the Jak-Stat pathway, which partly explains the regulation of miR-23a on lipid metabolism. This research provides novel information regarding the relationship between miR-23a and adipocytes.

## Figures and Tables

**Table 1:** Summary of the three DGE (digital gene expression) libraries.

	Control Cells	miR-23a Over-Expressed cells	miR-23a Knockdown Cells
Raw reads	9,011,507	10,563,298	9,726,783
Clean reads	8,994,528	10,538,070	9,706,883
Mapped reads	6,713,115	8,022,693	7,769,794
	74.64% ^a^	76.31% ^a^	80.04% ^a^
Match (uniqe Sense) ≤1 mismatch	6,585,804	7,866,468	7,626,414
	73.22% ^a^	74.35% ^a^	78.57% ^a^
Match (uniqe Antiense) ≤1 mismatch	29,777	37,633	31,751
	0.32% ^a^	0.36% ^a^	0.43% ^a^
Unmapped reads	2,281,413	2,515,377	1,937,089
	25.36% ^a^	23.87% ^a^	19.96% ^a^

^a^ represented the ratio from the comparison between the number of sequences and the number of clean reads.

## Supplementary Materials

The following are available online at www.mdpi.com/2073-4425/7/10/92/s1, Table S1. Primer sequences of qRT-PCR.
