# Supplementary material for: Effects of MicroRNA-23a on Differentiation and Gene Expression Profiles in 3T3-L1 Adipocytes
Source: Genes (Basel). 2016 Oct 24;7(10):92. doi: 10.3390/genes7100092 (PMC5083931; doi:10.3390/genes7100092)
Supplement: Supplementary file 1 [file genes-07-00092-s001.docx]

Supplementary Materials: Effects of MicroRNA-23a on Differentiation and Gene Expression Profiles in 3T3-L1 Adipocyte

Yong Huang, Jinxiu Huang, Renli Qi, Qi Wang, Yongjiang Wu and Jing Wang

**Table S1.** Primer sequences of qRT-PCR.

| **Genes** | **Primer Sequences (5’-3’)** |
| --- | --- |
| *miR-23a* | ATCACATTGCCAGGGATTTCC |
| *PPARγ* | F: CTCCAGCATTTCCACTCCAC |
|  | R: ACACAGGCTCCACTTTGATG |
| *C/EBPα* | F: GGCAAAGCCAAGAAGTCG |
|  | R: TGGTCAACTCCAGCACCTT |
| *FABP4* | F: GTCACCATCCGGTCAGAGA |
|  | R: TGATGCTCTTCACCTTCCTGT |
| *FAS* | F: GGCTGCTGTTGGAAGTCA |
|  | R: TGCCTCTGAACCACTCACAC |
